# Bare Neck and Arms

**Published:** 1877-04

**Authors:** 


**BARE NECK AND ARMS.**—There is, perhaps, not an eminent physician in any system of practice, who will not declare, with a distinguished medical practitioner, now deceased: "I believe, that during the twenty-six years I have followed my profession in this city, twenty thousand children have been carried to the cemeteries, a sacrifice to the absurd custom of exposing their arms naked."

*NETTLE RASH.*

PEN YAN, N. Y. March 11, 1858.

DR. STAFFORD: For eight years my wife had suffered almost daily with the Nettle Rash—so the doctor called it. The three packages of Iron and Sulpher Powders I sent for has cured her. She has had no itching for more than two months. Inclosed you will find one dollar for another package for a friend similarly afflicted; if they do as well as this case, you can sell lots of them in Yates County. Send by return mail.

Yours respectfully, SIMON G. ELBROCK.

Sold by Druggists. 1 Package, 12 Powders, $1; 6 Packages, 72 Powders, $5. Mailed free. Hall & Ruckel, 218 Greenwich Street, New York.

